# Efficacy and Safety of SARS-CoV-2 Neutralizing Antibody JS016 in Hospitalized Chinese Patients with COVID-19: a Phase 2/3, Multicenter, Randomized, Open-Label, Controlled Trial

**DOI:** 10.1128/aac.02045-21

**Published:** 2022-03-15

**Authors:** Run Dong, Li Jiang, Ting Yang, Changsong Wang, Yi Zhang, Xu Chen, Jianfeng Xie, Yuanbin Guo, Li Weng, Yan Kang, Kaijiang Yu, Haibo Qiu, Bin Du, for the China Critical Care Clinical Trials Group (CCCCTG)

**Affiliations:** a Medical Intensive Care Unit, State Key Laboratory of Complex Severe and Rare Diseases, Peking Union Medical College Hospital, Peking Union Medical College & Chinese Academy of Medical Sciences, Beijing, People’s Republic of China; b Department of Critical Care Medicine, Xuanwu Hospital, Capital Medical University, Beijing, People’s Republic of China; c Intensive Care Unit, Harbin Medical University Cancer Hospital, Harbin, Heilongjiang, People’s Republic of China; d Department of Critical Care Medicine, West China Hospital, Sichuan University, Chengdu, Sichuan, People’s Republic of China; e Intensive Care Unit, The First Affiliated Hospital of Harbin Medical University, Harbin, Heilongjiang, People’s Republic of China; f Department of Critical Care Medicine, Zhongda Hospital, Southeast University, Nanjing, Jiangsu, People’s Republic of China; g Intensive Care Unit, Shijiazhuang People’s Hospital, Shijiangzhuang, Hebei, People’s Republic of China

**Keywords:** COVID-19, SARS-CoV-2, neutralizing antibodies, JS016

## Abstract

Recombinant human severe acute respiratory syndrome coronavirus 2 (SARS-CoV-2) monoclonal antibody JS016 showed neutralizing and therapeutic effects in preclinical studies. The clinical efficacy and safety of the therapy needed to be evaluated. In this phase 2/3, multicenter, randomized, open-label, controlled trial, hospitalized patients with moderate or severe coronavirus disease 2019 (COVID-19) were randomly assigned in a 1:1 ratio to receive standard care or standard care plus a single intravenous infusion of JS016. The primary outcome was a six-level ordinal scale of clinical status on day 28 since randomization. Secondary outcomes include adverse events, 28-day mortality, ventilator-free days within 28 days, length of hospital stay, and negative conversion rate of SARS-CoV-2 nucleic acid on day 14. A total of 199 patients were randomized, and 197 (99 in the JS016 group and 98 in the control group) were analyzed. Most patients, 95 (96%) in the JS016 group and 97 (99%) in the control group were in the best category on day 28 since randomization. The odds ratio of being in a better clinical status was 0.31 (95% confidence interval [CI], 0.03 to 3.19; *P* = 0.33). Few adverse events occurred in both groups (3% in the JS016 group and 1% in the control group, respectively; *P* = 0.34). SARS-CoV-2 neutralizing antibody JS016 did not show clinical efficacy among hospitalized Chinese patients with moderate to severe COVID-19 disease. Further studies are needed to assess the efficacy of the neutralizing antibody to prevent disease deterioration and its benefits among groups of patients specified by disease course and severity. (This study has been registered at ClinicalTrials.gov under identifier NCT04931238.)

## INTRODUCTION

Since the first reports of coronavirus disease 2019 (COVID-19) caused by severe acute respiratory syndrome coronavirus 2 (SARS-CoV-2), the illness is still far from optimal control. The World Health Organization (WHO) declared the COVID-19 outbreak a global pandemic on March 11, 2020. Globally, as of September 2021, there have been more than 200 million confirmed cases, including more than 4 million deaths, reported to WHO. In total, 5 to 32% of patients required ICU admission, and mortality rates vary between 1.4 and 33% (1–9). However, except dexamethasone which has been shown to decrease mortality (10), there is a lack of additional effective and safe therapies.

Recombinant human SARS-CoV-2 monoclonal antibody JS016, obtained from a B lymphocyte of a COVID-19 survivor, binds with high-affinity to the receptor-binding domain within the S1 subunit of the SARS-CoV-2 spike protein, thus blocking the binding between the virus and the cell surface receptor angiotensin-converting enzyme 2 (ACE2). *In vitro* study and animal models showed prominent neutralizing and therapeutic effects of JS016 on SARS-CoV-2 infection (11). A phase 1 trial among healthy volunteers has demonstrated a tolerable and safe drug profile of JS016 (12). To evaluate the efficacy and safety of JS016 in patients hospitalized with COVID-19, we performed a phase 2/3, multicenter, randomized, open-label, controlled trial with the support of the China National Health Commission and the Ministry of Science and Technology.

## RESULTS

### Patient characteristics.

From January 18, 2021 to February 2, 2021, 199 patients were enrolled and randomized. A total of 100 patients were randomly assigned to receive standard care plus JS016, and 99 to receive standard care. After excluding patients receiving convalescent plasma (1 in JS016 group) or antiviral drugs (1 in the control group), 99 patients of the JS016 group and 98 of the control group entered final analyses. Patient characteristics at randomization were similar between the two groups (Table 1). The median age was 59, and 106 (54%) were male. There were 63 (32%) patients with coexisting diseases, including hypertension (25%), coronary heart disease (8%), diabetes (7%), and chronic pulmonary disease (3%). One hundred and 70 (86%) patients had moderate disease, and 27 (14%) had severe disease at randomization. Sixty-eight (35%) patients were from Hebei Province and 129 (65%) from Heilongjiang Province. Median days from symptom onset to randomization were 7, and median days from hospitalization to randomization were 4.

**TABLE 1:** Characteristics of the patients at randomization

Characteristics	JS016 (*n* = 99)	Control (*n* = 98)	Total (*n* = 197)
Male, n (%)	59 (60%)	47 (48%)	106 (54%)
Age, yr, median (IQR)	58 (47-68)	61 (51-67)	59 (50-67)
Body mass index, kg/m^2^, median (IQR)	23 (21-26)	25 (22-27)	24 (22-26)
Coexisting disease, n (%)			
Any	32 (32%)	31 (32%)	63 (32%)
Diabetes	6 (6%)	8 (8%)	14 (7%)
Hypertension	25 (25%)	25 (26%)	50 (25%)
Coronary heart disease	7 (7%)	9 (9%)	16 (8%)
Chronic pulmonary disease	4 (4%)	2 (2%)	6 (3%)
Chronic renal disease	0 (0%)	0 (0%)	0 (0%)
Cancer	0 (0%)	0 (0%)	0 (0%)
Site, n (%)			
Hebei	34 (34%)	34 (35%)	68 (35%)
Heilongjiang	65 (66%)	64 (65%)	129 (65%)
Days since symptom onset, median (IQR)	6 (5-9)	7 (4-9)	7 (5-9)
Days since hospitalization*, median (IQR)	4 (2-5)	3 (1-5)	4 (2-5)
Symptoms, n (%)			
Fever	32 (33%)	39 (40%)	71 (36%)
Cough	38 (39%)	31 (32%)	69 (35%)
Dyspnea	5 (5%)	3 (3%)	8 (4%)
Others	10 (10%)	11 (11%)	21 (11%)
Laboratory measures			
Lymphocytes, cells/mm^3^, median (IQR)	1240 (900-1630)	1210 (840-1600)	1230 (895-1605)
C-reactive protein*, mg/liter, median (IQR)	5.2 (1.0-26.7)	8.9 (1.2-34.0)	7.4 (1.1-31.9)
SARS-CoV-2 antibody, g/liter, median (IQR)			
IgM*	0.50 (0.10-4.30)	0.38 (0.10-2.96)	0.44 (0.10-3.68)
IgG	0.26 (0.08-5.20)	0.23 (0.06-2.90)	0.23 (0.07-4.52)
Oxygen requirement, n (%)			
None	63 (64%)	60 (62%)	123 (62.4%)
Nasal cannula	28 (28%)	34 (35%)	62 (31.5%)
Noninvasive ventilation or high flow nasal cannula	8 (8%)	3 (3%)	11 (5.6%)
Invasive ventilation or ECMO	0 (0%)	1 (1%)	1 (0.5%)
Severity, n (%)			
Moderate^*a*^	86 (87%)	84 (86%)	170 (86%)
Severe^*b*^	13 (13%)	14 (14%)	27 (14%)

*P < 0.05.

a Moderate illness was defined as fever or respiratory symptoms with pulmonary infiltration.

b Severe illness was defined if patients presented with any of the following conditions: (i) dyspnea or respiratory rate ≥30 per minute; (ii) arterial oxygen saturation ≤93% on room air at sea level; (iii) a ratio of arterial partial pressure of oxygen to fraction of inspired oxygen (PaO_2_/FiO_2_) ≤300mm Hg; (iv) progressive exacerbation of symptoms, and pulmonary infiltration progressing by more than 50 percent within 24 to 48 h.

All patients received traditional Chinese medicine, and a few patients received glucocorticoid (Table S2). Other baseline characteristics are in Table S1.

### Primary and secondary outcomes.

**FIG 1:**
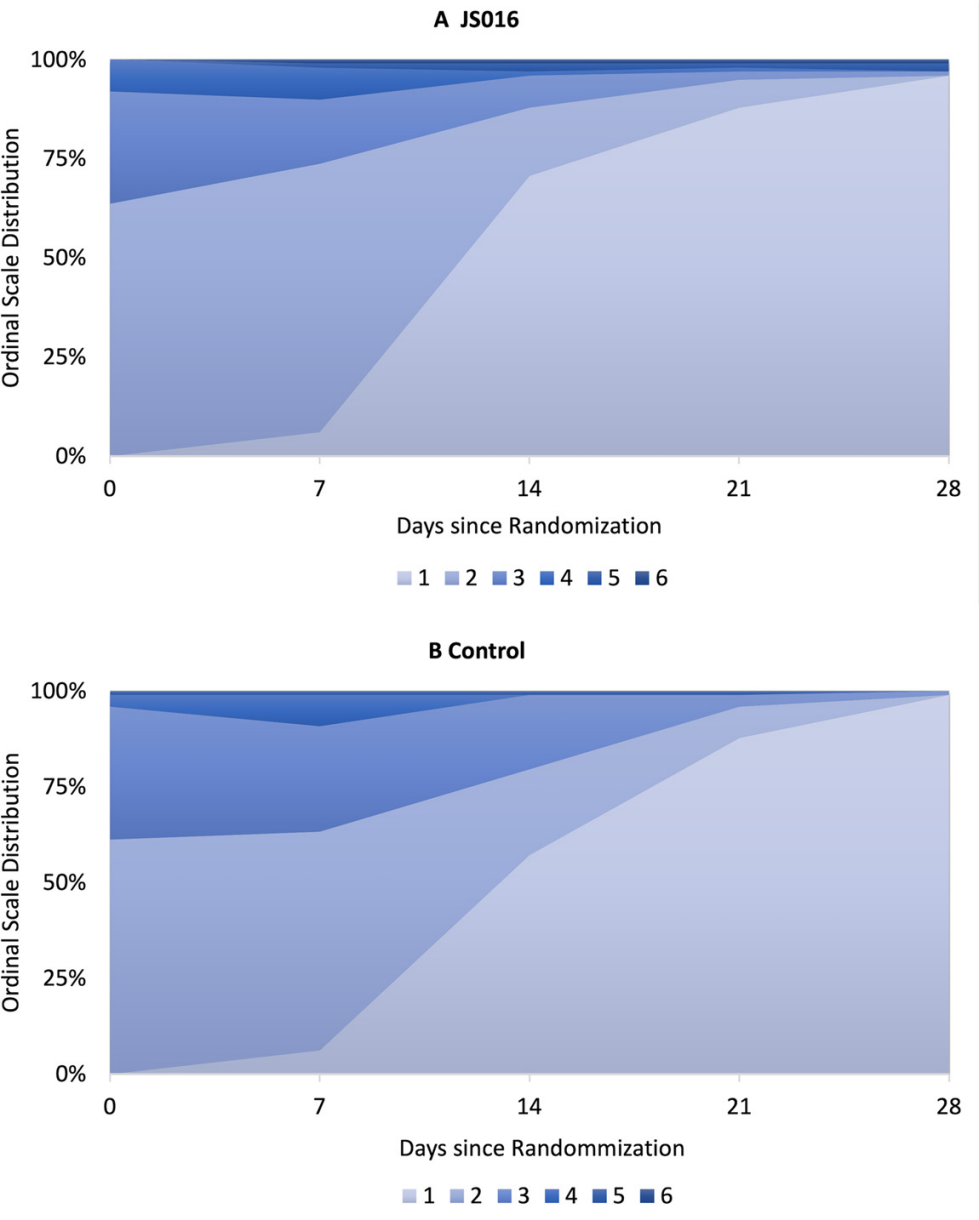
Distribution of ordinal scores over time since randomization. The 6-level ordinal scale of clinical status was defined as follows: a score of 1 indicated not hospitalized; 2, hospitalized without supplemental oxygen; 3, hospitalized with supplemental oxygen; 4, hospitalized with noninvasive ventilation or high flow nasal cannula; 5, hospitalized with invasive ventilation or ECMO; and 6, death.

Most patients, 95 (96%) in the JS016 group and 97 (99%) in the control group were in the best category on day 28 since randomization. The distribution of patients across the six-level ordinal scale was similar between the two groups. The odds ratio (OR) of being in a more favorable category in the JS016 group than in the control group was 0.31 (95% confidence interval [95% CI], 0.03 to 3.19; *P* = 0.33). The distribution of patients across the clinical ordinal scale were also similar between the two groups on day 7, 14, and 21. (Table 2, Fig. 1, and Table S3).

**TABLE 2:** Primary and secondary outcomes

Outcome	JS016 (*n* = 99)	Control (*n* = 98)	Comparison (95% CI)	*P* value
Primary outcome^*a*^			0.31 (0.03-3.19)	0.33
1	95 (96%)	97 (99%)		
2	0 (0%)	0 (0%)		
3	1 (1%)	1 (1%)		
4	0 (0%)	0 (0%)		
5	2 (2%)	0 (0%)		
6	1 (1%)	0 (0%)		
Secondary outcomes				
28-day mortality	1 (1%)	0 (0%)		0.99
Ventilator-free days within 28 days, median (IQR)	28 (28-28)	28 (28-28)	0.57 (0.27-1.24)	0.16
Length of hospital stay, days, median (IQR)	13 (11-15)	14 (11-17)	0.55 (0.13-2.31)	0.42
Negative conversion rate of SARS-CoV-2 nucleic acid on day 14^*b*^	82% (80/97)	78% (76/97)	1.35 (0.65-2.83)	0.43
ORF gene	82% (80/97)	79% (77/97)	1.27 (0.60-2.68)	0.53
N gene	82% (80/97)	78% (76/97)	1.35 (0.65-2.83)	0.43
Adverse events				
Any	3 (3%)	1 (1%)	3.16 (0.30-33.64)	0.34
Secondary infection	1 (1%)	0 (0%)		0.99
Elevated ALT or AST^*c*^	2 (2%)	1 (1%)	1.81 (0.15-22.57)	0.65
Acute kidney injury	1 (1%)	0 (0%)		0.99
Acute myocardial infarction	1 (1%)	0 (0%)		0.99
Allergic reaction	0 (0%)	0 (0%)		
Septic shock	0 (0%)	0 (0%)		
Gastrointestinal bleeding	0 (0%)	0 (0%)		

a A score of 1 indicated not hospitalized; 2, hospitalized without supplemental oxygen; 3, supplemental oxygen; 4, noninvasive ventilation or high flow nasal cannula; 5, invasive ventilation or ECMO; and 6, death.

b SARS-CoV-2 nucleic acid was defined as negative if both ORF and N gene turned negative.

c ALT, alanine transaminase; AST, aspartate transaminase.

There were no significant differences between the two groups in terms of 28-day mortality (*P* = 0.99), ventilator-free days within 28 days (OR, 0.57 [95% CI, 0.27 to 1.24]; *P* = 0.16), and length of hospital stay (OR, 0.55 [95% CI, 0.13 to 2.31]; *P* = 0.42) (Table 2).

Negative conversion rates of SARS-CoV-2 nucleic acid on day 14 since randomization were similar between two groups (OR, 1.35 [95% CI, 0.65 to 2.83]; *P* = 0.43), as well as on day 7 and 21 (Table 2 and Table S4). Cox proportional-hazards model showed no significant difference of the probability of negative conversion of SARS-CoV-2 nucleic acid (HR, 1.17 [95% CI, 0.88 to 1.55]; *P* = 0.23), ORF gene (HR, 1.03 [95% CI, 0.77 to 1.37]; *P* = 0.83), and N gene (HR, 1.17 [95% CI, 0.88 to 1.55]; *P* = 0.23) (Fig. S1).

Serum SARS-CoV-2 specific IgG was significantly higher in the JS016 group than in the control group from day 2 through day 14 since randomization. Serum-specific IgM was similar between the two groups across 21 days of follow-up. (Fig. S2 and Table S5)

Only 3 (3%) and 1 (1%) adverse events occurred in the JS016 group and the control group, respectively, including secondary infection, elevated liver enzymes, acute kidney injury, and acute myocardial infarction. There were no between-group differences in terms of the safety outcomes. (Table 2)

### Post hoc and subgroup analyses.

Post hoc analyses showed that JS016 group had lower risk of clinical status deterioration (8% versus 17%; OR, 0.37 [95% CI, 0.14 to 0.97]; *P* = 0.04) and development of supplemental oxygen requirement (2% versus 11%; OR, 0.16 [95% CI, 0.04 to 0.76]; *P* = 0.02) compared with control group.

Subgroup analyses among patients with moderate or severe illness, with or without supplemental oxygen requirement at randomization, and within or beyond 4 days since symptom onset, showed similar primary outcomes between the treatment and control group (Table S6). For patients without supplemental oxygen requirement at randomization, the negative conversion rate of SARS-CoV-2 nucleic acid on day 14 was higher among the JS016 group compared with the control group (90% versus 77%; OR, 2.90 [95% CI,1.03 to 8.16]; *P* = 0.04), and there was a trend of shorter hospital stay (12 versus 14 days; OR 0.19 [95% CI, 0.04 to 1.07]; *P* = 0.06). For patients within 4 days from symptom onset, JS016 group had higher negative conversion rate of SARS-CoV-2 nucleic acid on day 14 (92% versus 58%; OR, 22.58 [95% CI,1.03 to 493.31], *P* = 0.048) and shorter hospital stay (13 versus 18 days; OR, 0.01 [95% CI, 0 to 0.17]; *P* = 0.003) than control group.

## DISCUSSION

Our study showed that JS016, compared with the standard care group, did not generate better clinical outcomes among Chinese patients hospitalized with moderate or severe COVID-19 illness. Meanwhile, viral clearance was not improved by the neutralizing antibody.

Several neutralizing monoclonal antibodies targeting various epitopes on the SARS-CoV-2 spike protein are under investigation (13–15). Bamlanvimab (LY-CoV555) with or without the combination of etesevimab (LY-CoV06 or JS016) decreased viral load and risk of COVID-19 related hospitalization among outpatients compared with those who received placebo but did not improve clinical outcomes among hospitalized patients (16–19). Further analyses showed a potentially higher rate of sustained recovery among patients without intrinsic neutralizing antibodies at study entry (20). REGN-COV2, a neutralizing antibody cocktail of casirivimab (REGN10933) and imdevimab (REGN10987), improved viral clearance and possibly prevented hospitalization among nonhospitalized patients within 7 days since symptom onset, especially those who had an incompetent immune response or high viral load at baseline (21). For hospitalized patients without anti-spike IgG at enrollment, REGN-COV2 improved survival compared with the control group (22). According to the futility analysis of 217 patients requiring low-flow oxygen who were SARS-CoV-2 seronegative at baseline, REGN-COV2 was found to reduce viral load at day 11 and lower the risk of death or receiving mechanical ventilation compared with the placebo group (14).

Reasons for unfavorable efficacy outcomes might include the following. First, there is still no consensus on the optimal definition and timing of the outcome measurement for evaluating clinical benefit from COVID-19 treatment. The studied group of patients hospitalized with moderate to severe COVID-19 disease presented with a lower rate of supplemental oxygen requirement (38%) and coexisting diseases (32%), which are associated with mortality and severe illness, compared with previous studies (17, 22). Clinical outcomes such as clinical status on day 28 and mortality were too positive for this population to reveal the presence of clinical efficacy of the studied neutralizing antibody. Other studies among out-patients, who share several characteristics as stated above with our studied population, focused on the risk of disease exacerbation, i.e., COVID-19-related, medically attended visit (16, 18, 21). In our study, *post hoc* analyses showed that JS016 might reduce the risk of clinical deterioration and the development of supplemental oxygen requirements. Meanwhile, JS016 might potentially improve viral clearance and recovery rate among the subgroup without supplemental oxygen requirement at study entry, who largely overlap with ambulatory patients with moderate COVID-19 disease in other parts of the world. Second, the infection might have reached hyperinflammation status rather than the virus itself as the duration of symptoms extended, with the former unlikely to be dampened by neutralizing antibodies. Similar to the trial of bamlanivimab among hospitalized patients with COVID-19 (21), the median duration of symptoms at randomization was 7 days in our study. Both trials yielded no clinical efficacy of neutralizing antibody therapy. Nonetheless, nonhospitalized patients within 7 days since symptom onset seemed to benefit from neutralizing antibodies (16, 18, 19). In our study, subgroup analyses among patients within 4 days since symptom onset showed shorter hospital stay and higher negative conversion rate of SARS-CoV-2 nucleic acid for the JS016 group, suggesting potential virological and clinical efficacy of the neutralizing antibody among patients with early COVID-19. Third, patients with an incompetent immune response or high viral load might be more likely to benefit from neutralizing antibody therapy. The presence of intrinsic neutralizing antibodies was unknown in our study, but studies on bamlanivimab and REGN-COV2 both reported greater clinical benefits from neutralizing antibody treatment for patients without the presence of neutralizing antibodies at study entry, especially those with elevated antigen or viral RNA levels (20–22). Fourth, viruses may develop mutations leading to neutralizing antibody resistance either through natural evolution or due to the selection pressure of treatment (23, 24). We did not assess the prevalence and mutations of escape mutants in this trial, but another study on bamlanvimab monotherapy reported an increase of escape mutants in the intervention group compared with the placebo group (16). Viral mutations that could escape either casirivimab, imdevimab, or etesevimab were mapped and found to be present among circulating SARS-CoV-2 sequences (25). *In vitro* and human studies showed antibody cocktails targeting different epitopes of SARS-CoV-2 spike protein might prevent rapid mutational escape seen with individual antibodies (24, 26). Fifth, a low rate of penetration into the airway mucus may impair the efficacy of neutralizing antibodies (27, 28).

Our study was conducted before the emerging variants of SARS-CoV-2 circulated in China, but there is an urgent need to answer questions about the efficacy of neutralizing antibodies against the variants. *In vitro* studies showed that the neutralizing activity of JS016 retained for B.1.617 variant detected in India (i.e., delta) (29), but reduced or vanished for B.1.1.7 variant detected in the United Kingdom (i.e., alpha) (30, 31), B.1.351 in South Africa (i.e., beta) (30–32), and B.1.1.28 in Brazil (i.e., gamma) (31–33). *In vivo* studies are required to examine the potency of JS016 against current variants.

JS016 demonstrated a good safety profile in our study. None of the currently studied neutralizing antibodies were reported to have major safety concerns (15), which supports their anticipated safe use.

Our study has several limitations. First, we only enrolled hospitalized patients with moderate to severe disease. The efficacy and safety profiles could not be generalized to nonhospitalized patients with mild symptoms. Second, the rate of SARS-CoV-2 nucleic acid testing was relatively low. However, we imputed nucleic acid tests as negative if patients had already been discharged (Table S7) according to local practice. Third, we could not assess the immune response of patients at study entry and immunogenicity of JS016 since neutralizing antibody was not tested. Fourth, the trial was not blinded so the potential Hawthorne effect might have influenced clinical practice and decision making. Fifth, subgroup analyses were not prespecified, and the sample size was limited.

Our multicenter, open-label, randomized, controlled trial did not show clinical or virological efficacy of JS016 among Chinese hospitalized patients with moderate or severe COVID-19 illness. Potential benefits remain to be evaluated among groups of patients specified by disease course and severity. Further studies might be needed to assess the efficacy of the neutralizing antibody to prevent disease deterioration.

## MATERIALS AND METHODS

### Trial design.

The trial was designed by the executive committee and conducted at three hospitals in Hebei and Heilongjiang Province, China. The protocol was approved by ethics committees at the participating sites, and written informed consent was obtained from all the patients or their legally authorized representatives. The trial was funded by the National Health Commission and the Ministry of Science and Technology. The trial was overseen by an independent data and safety monitoring committee. The executive committee vouches for the completeness and accuracy of the data and the fidelity of the trial to the protocol. The trial was registered at ClincalTrials.gov (NCT04931238).

### Participants.

We enrolled 18 to 85-year-old patients who were hospitalized to participating hospitals with confirmed moderate or severe COVID-19 and who had a duration of symptoms of 7 days or less for moderate disease or duration of severe disease of 4 days or less. Definitions of disease severity were based on the Eighth Edition of Clinical Practice of COVID-19 issued by China National Health Commission (http://www.nhc.gov.cn/yzygj/s7653p/202104/7de0b3837c8b4606a0594aeb0105232b.shtml) and detailed in the protocol (Supplemental File 1). Patients were excluded if they had a critical disease or tested positive of SARS-CoV-2 specific immunoglobulin G (IgG) or immunoglobulin M (IgM) before enrollment. Other reasons for exclusion were class III or IV heart failure, left ventricular ejection fraction lower than 30%, confirmed or suspected active tuberculosis, chronic renal failure requiring renal replacement therapy, malignancy, pregnancy, and breastfeeding.

### Randomization, interventions, and data collection.

Patients were randomly assigned in a 1:1 ratio to receive standard care (control group) or standard care plus a single intravenous infusion of JS016, stratified by sites and disease severity at randomization (i.e., moderate or severe disease). A dose of 50 mg/kg was based on tolerability, safety, and pharmacokinetic data (12). The standard care was based on the Eighth Edition of Clinical Practice of COVID-19 issued by China National Health Commission (http://www.nhc.gov.cn/yzygj/s7653p/202104/7de0b3837c8b4606a0594aeb0105232b.shtml). Other medications were allowed except for those providing exogenous antibodies against SARS-CoV-2. Data were collected on randomization and day 7, 14, 21, and 28 since randomization.

### Outcomes.

The primary outcome was a six-level ordinal scale of clinical status on day 28 since randomization (34–36), which was defined as follows: a score of 1 indicated not hospitalized; 2, hospitalized without supplemental oxygen; 3, hospitalized with supplemental oxygen; 4, hospitalized with noninvasive ventilation or high flow nasal cannula; 5, hospitalized with invasive ventilation or extracorporeal membrane oxygenation (ECMO); and 6, death.

Secondary outcomes included 28-day mortality, ventilator-free days within 28 days, length of hospital stay, negative conversion rate of SARS-CoV-2 nucleic acid on day 14. Safety outcomes included allergic reaction, secondary infection, elevated alanine or aspartate transaminase (ALT or AST), acute kidney injury, acute myocardial infarction, septic shock, and gastrointestinal bleeding.

### Statistical analyses.

According to the distribution across the six-level ordinal outcome of another study among similar patients (37), we calculated that the initial enrollment of 200 patients would have 80% power to detect an odds ratio of 1.50 for a better outcome category with a one-sided type I error of 0.3. We chose a high type I error rate to prevent enrolling too many patients in an investigational drug that is unlikely to be effective.

To eliminate the confounding effect of antibody-based and antiviral therapy, patients receiving convalescent plasma (1 in the treatment group) or antiviral drugs (1 in the control group) were also excluded from the final analyses.

Post hoc analyses were performed evaluating deterioration of clinical status, defined as an increase of the six-level ordinal scale of clinical status through 28 days from randomization, and development of supplemental oxygen requirement. Subgroup analyses were also conducted among patients with moderate or severe illness, with or without supplemental oxygen requirement at study entry, and within or beyond 4 days since symptom onset.

The primary outcome was analyzed by mixed ordinal logistic regression. Binary outcomes were compared with the use of a mixed logistic regression model. 28-day mortality and negative conversion rates of SARS-CoV-2 nucleic acid were analyzed by the Cox proportional-hazards model. Continuous outcomes were assessed with the use of generalized linear models or general linear models for repeated measures. All models were adjusted for age, site, and clinical ordinal scale at randomization. *P* value less than 0.05 was considered statistically significant. Statistical analyses were performed by SPSS 22.0.
