# Supplementary material for: Efficacy and Safety of SARS-CoV-2 Neutralizing Antibody JS016 in Hospitalized Chinese Patients with COVID-19: a Phase 2/3, Multicenter, Randomized, Open-Label, Controlled Trial
Source: Antimicrob Agents Chemother. 2022 Mar 15;66(3):e02045-21. doi: 10.1128/aac.02045-21 (PMC8923168; doi:10.1128/aac.02045-21)
Supplement: Supplemental file 1 — Supplemental material. Download aac.02045-21-s0001.pdf, PDF file, 0.5 MB [file aac.02045-21-s0001.pdf]

Supplemental Material

Table S1. Additional characteristics of the patients at randomization 2
Table S2. Treatment and Supportive Care..2
Table S3. Six-level ordinal outcomes on day 7, 14 and 21 since randomization 3
Table S4. Negative conversion rate of SARS-CoV-2 nucleic acid on day 7, 14 and 21 4
Figure S1. Probability of negative conversion of SARS-CoV-2 nucleic acid, ORF gene, and N gene...5
Table S5. Serum SARS-CoV-2 Specific Antibody from Randomization to Day 21 6
Figure S2. Serum SARS-CoV-2 Specific Immunoglobulin...7
Table S6. Subgroup analyses...8
Table S7. Imputation of Missing Data for SARS-CoV-2 Nucleic Acid Tests..............................11
Protocol..12

Table S1. Additional characteristics of the patients at randomization

Characteristics	JS016 (n = 99)	Control (n = 98)	Total (n = 197)
Vital signs			
Temperature, ℃, median (IQR)	36.6 (36.1-37.1)	36.8 (36.4-37.8)	36.7 (36.3-37.3)
SBP, mmHg, mean ± SD	124 ± 18	125 ± 17	125 ± 17
DBP, mmHg, mean ± SD	77 ± 12	77 ± 11	77 ± 11
Heart rate, bpm, median (IQR)	76 (70-84)	82 (74-92)	80 (70-90)
Respiratory rate, /min, median (IQR)	18 (18-20)	18 (18-20)	18 (18-20)
SpO2, %, median (IQR)	97 (96-98)	97 (97-98)	97 (96-98)
Laboratory measures			
pH, median (IQR)	7.43 ± 0.04	7.44 ± 0.05	7.43 ± 0.04
PaO_2*, mmHg, median (IQR)	92 (81-125)	77 (70-93)	84 (74-99)
$PaCO_2$, mmHg, mean ± SD	37 ± 5	36 ± 5	37 ± 5
HCO_3, mmol/L, mean ± SD	24 ± 3	24 ± 3	24 ± 3
White cell count, cells/mm^3, median (IQR)	4800 (3920-5690)	4705 (3880-5980)	4770 (3900-5900)
Platelet count, 10^3 cells/mm^3, median (IQR)	172 (144-228)	174 (136-216)	173 (139-221)
Total bilirubin, mg/dL, median (IQR)	0.49 (0.35-0.69)	0.54 (0.36-0.68)	0.51 (0.35-0.68)
LDH, U/L, median (IQR)	230 (158-428)	239 (168-465)	236 (163-454)
ALT, U/L, median (IQR)	21 (14-38)	25 (16-36)	23 (15-37)
Creatinine, mg/dL, median (IQR)	0.70 (0.59-0.81)	0.67 (0.57-0.80)	0.68 (0.58-0.81)

*$p<0.05$

SBP, systolic blood pressure; DBP, diastolic blood pressure; LDH, lactate dehydrogenase; ALT, alanine aminotransferase.

Table S2. Treatment and Supportive Care

Treatment or supportive care	JS016 (n = 99)	Control (n = 98)	Total (n = 197)
Medication			
Traditional Chinese medicine	99 (100%)	98 (100%)	197 (100%)
Glucocorticoid	1 (1%)	2 (2%)	3 (1.5%)
Supportive care			
Supplemental oxygen	36 (36%)	39 (40%)	75 (38%)
Noninvasive ventilation	4 (4%)	0 (1%)	4 (2%)
ECMO	1 (1%)	1 (1%)	2 (1%)
Vasopressor	1 (1%)	1 (1%)	2 (1%)
Renal replacement therapy	0 (0%)	1 (1%)	1 (0.5%)

Table S3. Six-level ordinal outcomes on day 7, 14 and 21 since randomization

Outcome [a]	JS016 (n = 99)	Control (n = 98)	Odds ratio (95% CI)	p
Day 7			1.31 (0.70-2.45)	0.40
1	6 (6%)	6 (6%)		
2	67 (68%)	56 (57%)		
3	16 (16%)	27 (28%)		
4	8 (8%)	8 (8%)		
5	1 (1%)	1 (1%)		
6	1 (1%)	0 (0%)		
Day 14			1.70 (0.93-3.09)	0.08
1	70 (71%)	56 (57.2%)		
2	17 (17%)	22 (22.4%)		
3	8 (8%)	19 (19.4%)		
4	1 (1%)	0 (0%)		
5	2 (2%)	1 (1%)		
6	1 (1%)	0 (0%)		
Day 21			0.85 (0.35-2.06)	0.71
1	87 (88%)	86 (88%)		
2	7 (7%)	8 (8%)		
3	2 (2%)	3 (3%)		
4	1 (1%)	0 (0%)		
5	1 (1%)	1 (1%)		
6	1 (1%)	0 (0%)		

a A score of 1 indicated not hospitalized; 2, hospitalized without supplemental oxygen; 3, supplemental oxygen; 4, noninvasive ventilation or high flow nasal cannula; 5, invasive ventilation or ECMO; and 6, death. ALT, alanine transaminase. AST, aspartate transaminase.

Table S4. Negative conversion rate of SARS-CoV-2 nucleic acid on day 7, 14 and 21

Outcome	JS016 (n = 99)	Control (n = 98)	Odds ratio (95% CI)	p
Negative conversion rate of SARS-CoV-2 nucleic acid on day 7 [a]	19% (18/97)	10% (10/97)	2.29 (0.93-5.65)	0.07
ORF gene	20% (19/97)	24% (23/97)	0.73 (0.33-1.62)	0.43
N gene	19% (18/97)	10% (10/97)	2.29 (0.93-5.65)	0.07
Negative conversion rate of SARS-CoV-2 nucleic acid on day 14 [a]	82% (80/97)	78% (76/97)	1.35 (0.65-2.83)	0.43
ORF gene	82% (80/97)	79% (77/97)	1.27 (0.60-2.68)	0.53
N gene	82% (80/97)	78% (76/97)	1.35 (0.65-2.83)	0.43
Negative conversion rate of SARS-CoV-2 nucleic acid on day 21 [a]	99% (96/97)	94% (91/97)	5.93 (0.69-50.77)	0.10
ORF gene	99% (96/97)	96% (93/97)	4.13 (0.44-38.51)	0.21
N gene	99% (96/97)	94% (91/97)	5.93 (0.69-50.77)	0.10

a SARS-CoV-2 nucleic acid was defined as negative if both ORF and N gene turned negative.

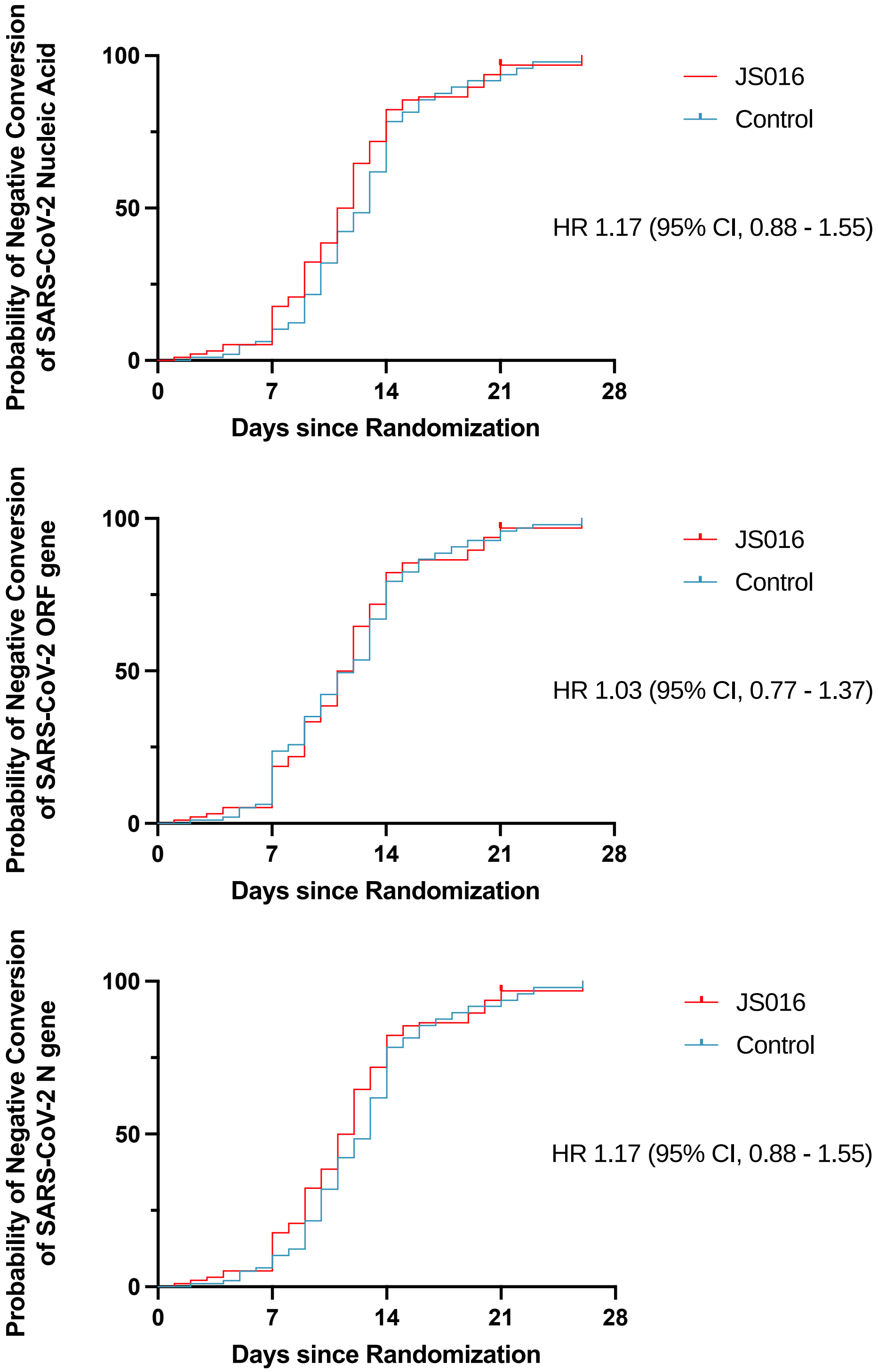

Figure S1. Probability of negative conversion of SARS-CoV-2 nucleic acid, ORF gene, and N gene. Hazard ratios (HR) were estimated using log-rank test of Cox proportional-hazards model, adjusted for age, site, and six-level ordinal scale of clinical status at randomization.

Table S5. Serum SARS-CoV-2 Specific Antibody from Randomization to Day 21

	JS016<br>(n = 99)	Control<br>(n = 98)	P
At randomization			
IgG	0.26 (0.08-5.20)<br>(n = 85)	0.23 (0.06-2.90)<br>(n = 86)	0.14
IgM	0.50 (0.10-4.30)<br>(n = 85)	0.38 (0.10-2.96)<br>(n = 86)	0.11
Day 2			
IgG	388.20 (376.65-398.34)<br>(n = 87)	6.92 (1.62-26.26)<br>(n = 77)	< 0.001
IgM	5.83 (1.29-14.31)<br>(n = 86)	4.28 (0.77-10.74)<br>(n = 77)	0.09
Day 7			
IgG	390.97 (377.43-399.64)<br>(n = 93)	32.34 (11.90-141.51)<br>(n = 90)	0.01
IgM	10.78 (3.85-25.36)<br>(n = 93)	11.96 (5.31-31.08)<br>(n = 90)	0.26
Day 14			
IgG	403.35 (398.09-417.15)<br>(n = 38)	102.45 (33.11-255.63)<br>(n = 53)	0.04
IgM	13.69 (4.46-30.39)<br>(n = 39)	17.64 (7.42-34.88)<br>(n = 53)	0.98
Day 21			
IgG	398.53 (395.41-402.06)<br>(n = 9)	172.72 (59.33-266.49)<br>(n = 13)	0.05
IgM	12.70 (1.96-23.45)<br>(n = 9)	22.07 (11.70-27.65)<br>(n = 13)	0.16

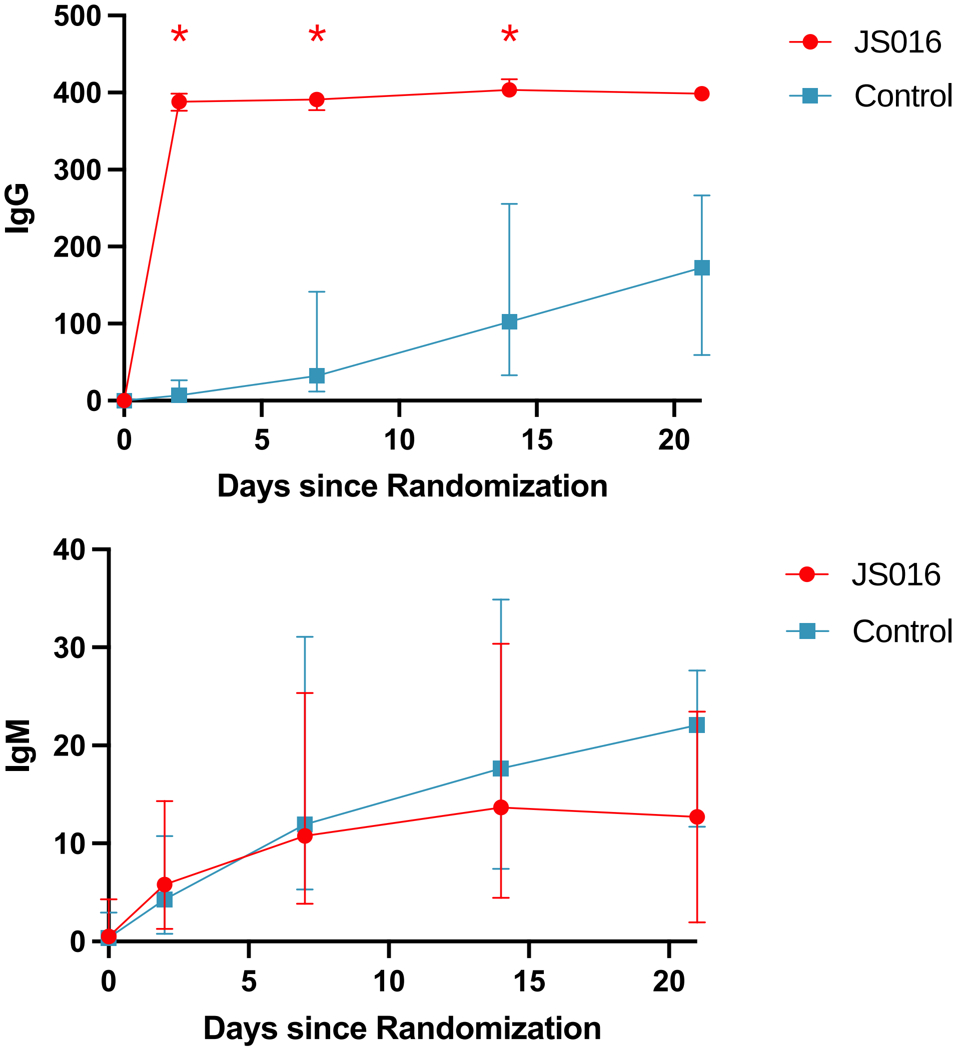

Figure S2. Serum SARS-CoV-2 Specific Immunoglobulin. Serum SARS-CoV-2 specific IgG and IgM was compared between the JS016 group and the control group using general linear models for repeated measures, adjusted for age, site, and six-level ordinal scale of clinical status at randomization. * $p < 0.05$.

Table S6. Subgroup analyses

Outcome	JS016	Control	Comparison (95% CI)	p
**Moderate disease**				
Number of patients, n/N	86/99	84/98		
Primary outcome [a]				0.99
1	83 (97%)	84 (100%)		
2	0 (0%)	0 (0%)		
3	1 (1%)	0 (0%)		
4	0 (0%)	0 (0%)		
5	1 (1%)	0 (0%)		
6	1 (1%)	0 (0%)		
Secondary outcomes				
28-day mortality	1 (1%)	0 (0%)		0.99
Ventilator-free days within 28 days, median (IQR)	28 (28-28)	28 (28-28)	0.74 (0.35-1.57)	0.44
Length of hospital stay, days, median (IQR)	13 (11-16)	12 (10-14)	0.45 (0.10-2.08)	0.31
Negative conversion rate of SARS-CoV-2 nucleic acid on day 14 [b]	85% (72/85)	79% (66/84)	1.50 (0.66-3.38)	0.33
ORF gene	85% (72/85)	80% (67/84)	1.38 (0.60-3.16)	0.45
N gene	85% (72/85)	79% (66/84)	1.50 (0.66-3.38)	0.33
Adverse events	3 (3%)	0 (0%)		0.99
**Severe disease**				
Number of patients, n/N	13/99	14/98		
Primary outcome [a]			1.82 (0.07-49.05)	0.72
1	12 (92%)	13 (93%)		
2	0 (0%)	0 (0%)		
3	0 (0%)	0 (0%)		
4	0 (0%)	1 (7%)		
5	1 (8%)	0 (0%)		
6	0 (0%)	0 (0%)		
Secondary outcomes				
28-day mortality	0 (0%)	0 (0%)		
Ventilator-free days within 28 days, median (IQR)	28 (28-28)	28 (28-28)	0.25 (0.01-5.16)	0.37
Length of hospital stay, days, median (IQR)	14 (13-21)	16 (14-19)	1.13 (0.04-35.36)	0.94
Negative conversion rate of SARS-CoV-2 nucleic acid on day 14 [b]	67% (8/12)	77% (10/13)	1.40 (0.15-12.92)	0.77
ORF gene	67% (8/12)	77% (10/13)	1.40 (0.15-12.92)	0.77
N gene	67% (8/12)	77% (10/13)	1.40 (0.15-12.92)	0.77
Adverse events	0 (0%)	1 (7%)		0.99

Outcome	JS016	Control	Comparison (95% CI)	p
**No supplemental oxygen requirement at randomization**				
Number of patients, n/N	63/99	60/98		
Primary outcome [a]				0.99
1	62 (98%)	60 (100%)		
2	0 (0%)	0 (0%)		
3	1 (2%)	0 (0%)		
4	0 (0%)	0 (0%)		
5	0 (8%)	0 (0%)		
6	0 (0%)	0 (0%)		
Secondary outcomes				
28-day mortality	0 (0%)	0 (0%)		
Ventilator-free days within 28 days, median (IQR)	28 (28-28)	28 (28-28)		1.0
Length of hospital stay, days, median (IQR)	12 (10-14)	14 (11-17)	0.19 (0.04-1.07)	0.06
Negative conversion rate of SARS-CoV-2 nucleic acid on day 14 [b]	90% (57/63)	77% (46/60)	2.90 (1.03-8.16)	0.04
ORF gene	90% (57/63)	78% (47/60)	2.64 (0.93-7.52)	0.07
N gene	90% (57/63)	77% (46/60)	2.90 (1.03-8.16)	0.04
Adverse events	1 (2%)	0 (0%)		0.96
**Supplemental oxygen requirement at randomization**				
Number of patients, n/N	36/99	38/98		
Primary outcome [a]			0.72 (0.04-12.07)	0.82
1	33 (92%)	37 (97%)		
2	0 (0%)	0 (0%)		
3	0 (0%)	0 (0%)		
4	0 (0%)	1 (3%)		
5	2 (5%)	0 (0%)		
6	1 (3%)	0 (0%)		
Secondary outcomes				
28-day mortality	1 (3%)	0 (0%)		0.99
Ventilator-free days within 28 days, median (IQR)	28 (28-28)	28 (28-28)	0.21 (0.03-1.59)	0.13
Length of hospital stay, days, median (IQR)	14 (12-21)	14 (11-17)	3.15 (0.31-32.25)	0.34
Negative conversion rate of SARS-CoV-2 nucleic acid on day 14 [b]	68% (23/34)	81% (30/37)	0.49 (0.15-1.62)	0.24
ORF gene	68% (23/34)	81% (30/37)	0.49 (0.15-1.62)	0.24
N gene	68% (23/34)	81% (30/37)	0.49 (0.15-1.62)	0.24
Adverse events	2 (5%)	1 (3%)	2.51 (0.16-39.13)	0.51

Outcome	JS016	Control	Comparison (95% CI)	p
**≤ 4 days since symptom onset**				
Number of patients, n/N	13/99	19/98		
Primary outcome [a]				1.0
1	13 (100%)	18 (95%)		
2	0 (0%)	0 (0%)		
3	0 (0%)	0 (0%)		
4	0 (0%)	1 (5%)		
5	0 (0%)	0 (0%)		
6	0 (0%)	0 (0%)		
Secondary outcomes				
28-day mortality	0 (0%)	0 (0%)		
Ventilator-free days within 28 days, median (IQR)	28 (28-28)	28 (28-28)		1.0
Length of hospital stay, days, median (IQR)	13 (11-14)	18 (14-22)	0.01 (0-0.17)	0.003
Negative conversion rate of SARS-CoV-2 nucleic acid on day 14 [b]	92% (12/13)	61% (11/18)	22.58 (1.03-493.31)	0.048
ORF gene	92% (12/13)	61% (11/18)	22.58 (1.03-493.31)	0.048
N gene	92% (12/13)	61% (11/18)	22.58 (1.03-493.31)	0.048
Adverse events	0 (0%)	0 (0%)		
**> 4 days since symptom onset**				
Number of patients, n/N	59/99	52/98		
Primary outcome [a]				0.99
1	55 (93%)	52 (100%)		
2	0 (0%)	0 (0%)		
3	1 (2%)	0 (0%)		
4	0 (0%)	0 (0%)		
5	2 (3%)	0 (0%)		
6	1 (2%)	0 (0%)		
Secondary outcomes				
28-day mortality	1 (2%)	0 (0%)		0.99
Ventilator-free days within 28 days, median (IQR)	28 (28-28)	28 (28-28)	0.34 (0.09-1.33)	0.12
Length of hospital stay, days, median (IQR)	13 (11-14)	18 (14-22)	2.16 (0.33-14.26)	0.42
Negative conversion rate of SARS-CoV-2 nucleic acid on day 14 [b]	81% (46/57)	85% (44/52)	0.69 (0.23-2.06)	0.51
ORF gene	81% (46/57)	87% (45/52)	0.56 (0.18-1.76)	0.32
N gene	81% (46/57)	85% (44/52)	0.69 (0.23-2.06)	0.51
Adverse events	2 (3%)	1 (2%)	2.36 (0.18-31.77)	0.52

a A score of 1 indicated not hospitalized; 2, hospitalized without supplemental oxygen; 3, supplemental oxygen; 4, noninvasive ventilation or high flow nasal cannula; 5, invasive ventilation or ECMO; and 6, death.

b SARS-CoV-2 nucleic acid was defined as negative if both ORF and N gene turned negative.

Table S7. Imputation of Missing Data for SARS-CoV-2 Nucleic Acid Tests

SARS-CoV-2 Nucleic acid tests	Original data missing	Data imputation [a]	Data missing in final analysis
Day 7			
ORF gene	55% (109/197)	54% (106/197)	1.5% (3/197)
N gene	46% (91/197)	45% (88/197)	1.5% (3/197)
Day 14			
ORF gene	84% (165/197)	82% (162/197)	1.5% (3/197)
N gene	84% (165/197)	82% (162/197)	1.5% (3/197)
Day 21			
ORF gene	95% (188/197)	94% (185/197)	1.5% (3/197)
N gene	94% (185/197)	92% (182/197)	1.5% (3/197)

[a] Since one of the requisites for discharge was two consecutive negative tests of SARS-CoV-2 nucleic acid, nucleic acid tests were imputed to be negative if the patient had already been discharged.

Protocol

Efficacy and Safety of a Recombinant Neutralizing Human Anti-SARS-CoV-2 Monoclonal Antibody (JS016) in Hospitalized Chinese Patients with SARS-CoV-2 Infection (COVID-19)

Short title: Efficacy and Safety of JS016 in COVID-19 Patients

Version: 1.0, 15 Jan 2021

NCT04931238

Funded by China National Health Commission and the Ministry of Science and Technology

Investigators and Affilliations:

Medical Intensive Care Unit, State Key Laboratory of Complex Severe and Rare Diseases, Peking Union Medical College Hospital, Peking Union Medical College & Chinese Academy of Medical Sciences, Beijing, People’s Republic of China (Run Dong, Ting Yang, Li Weng, Bin Du).
Department of Critical Care Medicine, Zhongda Hospital, Southeast University, Nanjing, Jiangsu, People’s Republic of China (Haibo Qiu, Jianfeng Xie).
Intensive Care Unit, The First Affiliated Hospital of Harbin Medical University, Harbin, Heilongjiang, People’s Republic of China (Kaijiang Yu, Xu Chen)
Department of Critical Care Medicine, Xuanwu Hospital, Capital Medical University, Beijing, People’s Republic of China (Li Jiang).
Department of Critical Care Medicine, West China Hospital, Sichuan University, Chengdu, Sichuan, People’s Republic of China (Yan Kang, Yi Zhang).
Intensive Care Unit, Harbin Medical University Cancer Hospital, Harbin, Heilongjiang, People’s Republic of China (Changsong Wang).
Intensive Care Unit, Shijiazhuang People’s Hospital, Shijiangzhuang, Hebei, People’s Republic of China (Yuanbin Guo).
For the China Critical Care Clinical Trials Group (CCCCTG)

Responsibilities:

Concept and design: BD
Data collection: LJ, YZ, YG, JX, XC, CW, TY
Statistical analyses and result interpretation: RD, TY, LW, BD
Study supervision: LW, LJ, HQ, KY, YK, BD
Report drafting: RD, LW, BD

1. Protocol Summary

1.1 Rationale

Coronavirus disease 2019 (COVID-19), caused by severe acute respiratory syndrome coronavirus 2 (SARS-CoV-2), has led to a global pandemic including millions of critical cases and deaths. There is an urgent need for effective and safe therapies. JS016 is a recombinant neutralizing human SARS-CoV-2 monoclonal antibody binding to SARS-CoV-2 S protein, and thus provides potential therapeutic effects by blocking the attachment and entry of the virus into human cells. This study aims to evaluate the efficacy and safety of JS016 in patients hospitalized with COVID-19. The data from this study will inform decisions of the clinical use of JS016.

1.2 Design

This is a multicenter, randomized, open-label, controlled trial to evaluate the efficacy and safety of JS016 in patients hospitalized with COVID-19.

Patients hospitalized to participating hospitals with moderate to severe COVID-19 will be randomized to receive standard care or standard care plus a single intravenous dose of JS016, and followed up for 28 days after randomization. The primary outcome, for clinical efficacy evaluation, is a six-level ordinal scale of clinical status on day 28 since randomization. Secondary outcomes include safety outcomes and other endpoints reflecting clinical efficacy. We plan to enroll a sample size of 200 participants. There will be a Data and Safety Monitoring Board.

2. Background and Study Rationale

Since the first reports of coronavirus disease 2019 (COVID-19) caused by severe acute respiratory syndrome coronavirus 2 (SARS-CoV-2), the illness is still far from optimal control. The World Health Organization (WHO) declared COVID-19 outbreak a global pandemic on March 11, 2020. Glogally, as of Jan 2021, there have been more than 100 million confirmed cases, including more than 2.5 million deaths, reported to WHO. 5-32% of patients required ICU admission, and mortality rates varies between 1.4-33%[1–9]. However, except dexamethasone which has been shown to decrease mortality[10], there is a lack of additional effective and safe therapies.

The SARS-CoV-2 enters host cells through binding of the spike (S) protein to the cell surface receptor angiotensin-converting enzyme 2 (ACE2)[11]. As the crucial component of viral surface, the S protein can be further separated into S1 and S2 subdomains, with S1 binding to the host receptor and S2 mediating membrane fusion. The receptor-binding domain (RBD) at the S1 C-terminal domain engages cell surface ACE2 receptor. Once in the cell, the virus completes genome replication, protein translation, assembly, and shedding.

Recombinant neutralizing human SARS-CoV-2 monoclonal antibody JS016, obtained from a B lymphocyte of a COVID-19 survivor, binds with high affinity to the RBD within the S1 subdomain of the SARS-CoV-2 S protein, thus blocks the binding between the virus and the cell surface receptor ACE2. The blocking of viral entry into cells is expected to relieve symptoms, mitigate the severity of disease, and improve clinical outcomes in patients infected with COVID-19.

In vitro study and animal models showed potent neutralizing and therapeutic effects of JS016 on SARS-CoV-2 infection[12]. Two phase I trials among healthy volunteers has demonstrated a tolerable and safe drug profile of JS016.

This study aims to evaluate the efficacy and safety of JS016 in patients hospitalized with COVID-19. The data from this study will inform decisions of the clinical use of JS016.

3. Study Design

This is a phase 2/3, multicenter, randomized, open-label, controlled trial to evaluate the efficacy and safety of JS016 in patients hospitalized with moderate or severe COVID-19 illness.

3.1 Pariticpating Hospitals

The First Affliated Hospital of Harbin Medical University, Heilongjiang, China
The First Hospital of Suihua, Heilongjiang, China
Shijiazhuang People's Hospital, Hebei, China

3.2 Study Population

3.2.1 Inclusion Criteria

Participants are eligible to be included in the study only if all of the following criteria apply:

1. Between 18 to 85 years of age at the time of randomization
2. With confirmed moderate or severe SARS-CoV-2 infection (COVID-19) according to Eighth Edition of Clinical Practice of COVID-19 issued by China National Health Commission[13]
3. Within 7 days since symptom onset or 4 days since the development of severe illness

3.2.2 Exclusion Criteria

Participants are excluded from the study if any of the following criteria apply:

1. Critical COVID-19 illness
2. Tested positive of SARS-CoV-2 specific antibodies, including immunoglobulin G (IgG) and immunoglobulin M (IgM) before enrollment
3. Class III or IV heart failure, or left ventricular ejection fraction lower than 30 percent
4. Confirmed or suspected active tuberculosis
5. Chronic renal failure requiring renal replacement therapy
6. Malignancy, including solid and hematologic malignancy
7. Pregnant or breastfeeding

3.2.3 Definitions

Suspected case of SARS-CoV-2 infection is defined as a person who meets:
One or more epidemiological criteria and two or more clinical criteria
OR
Two or more clinical criteria and a positive test of SARS-CoV-2 specific immunoglobulin M (IgM)
OR
All of the three clinical criteria, in the absence of epidemiological history
Epidemiological Criteria:
Residing or travel to a community with COVID-19 case any time within the 14 days prior to symptom onset
Contact of SARS-CoV-2 infected patients any time within the 14 days prior to symptom onset

Contact of patients with fever or respiratory tract symptoms from a community with COVID-19 case any time within the 14 days prior to symptom onset
Cluster onset, i.e., two or more cases with fever and/or respiratory tract symptoms in settings like family, office, or class, within 14 days
Clinical Criteria:
Fever and/or COVID-19 related symptoms
Typical chest imaging findings suggestive of COVID-19
Normal or decreased white blood cell and lymphocyte count

Comfirmed case of SARS-CoV-2 infection is defined as a suspected case with a positive pathogenic or serologic test, including:
A positive RT-PCR test of SARS-CoV-2 nucleic acid
Viral genetic sequence highly homologous to the known sequence of SARS-CoV-2
A positive test of SARS-CoV-2 specific antibodies, including IgM and IgG
Positive conversion of SARS-CoV-2 specific IgG, an increase of IgG titer by four times or more

Disease Severity:
Moderate illness is defined as fever or respiratory tract symptoms with pulmonary infiltration.
Severe illness is diagnosed if a patient presents with any of the following conditions:
1. Dyspnea or respiratory rate ≥ 30 per minute
2. Arterial oxygen saturation ≤ 93% on room air at sea level
3. A ratio of arterial partial pressure of oxygen to fraction of inspired oxygen (PaO_2/FiO_2) ≤ 300mmHg
4. Progressive worsening of symptoms, and pulmonary infiltration progressing by more than 50 percent within 24 to 48 hours.

Critical illness is diagnosed if a patient develops respiratory failure requiring mechanical ventilation, shock, and/or multiple organ dysfunction requiring ICU admission.

3.3 Intervention
Participants are randomly assigned in a 1:1 ratio to receive standard care (control group) or standard care plus a single intravenous infusion of JS016, stratified by sites and disease severity at randomization (i.e., moderate or severe illness). A dose of 50mg/kg is based on tolerability, safety, and pharmacokinetic data. The standard care, based on the Eighth Edition of Clinical Practice of COVID-19 issued by China National Health Commission, includes monitoring, supplemental oxygen, respiratory support, and other supportive therapies[13]. Other medications are allowed except for those providing exogenous antibodies against SARS-CoV-2.

3.4 Objectives and outcomes

Objectives	Outcomes
Primary	
Efficacy of JS016 on clinical status	Six-level ordinal scale of clinical status on 28 days since randomization[14–16] (**eTable 1**)
Secondary	

Efficacy of JS016 on clinical outcomes	Mortality rate within 28 days since randomization<br>Ventilator free days within 28 days since randomization<br>Length of hospital stay
Efficacy of JS016 on viral clearance	Negative conversion rate of SARS-CoV-2 nucleic acid on day 14 since randomization.<br>Negative conversion rate of SARS-CoV-2 nucleic acid on day 7 and 21 will also be recorded. SARS-CoV-2 nucleic acid was defined as negative if both ORF and N gene turned negative.
Safety assessment	Adverse events (AEs) including allergic reaction, secondary infection, elevated alanine or aspartate transaminase (ALT or AST), acute kidney injury, acute myocardial infarction, septic shock, and gastrointestinal bleeding

eTable 1. The primary outcome: six-level ordinal scale of clinical status

Score	Clinical status
1	Not hospitalized and without supplemental oxygen
2	Hospitalized without supplemental oxygen
3	Hospitalized with supplemental oxygen
4	Hospitalized with noninvasive ventilation or high flow nasal cannula
5	Hospitalized with invasive ventilation or ECMO
6	Death

3.5 Data Collection

Data are collected on randomization and day 7, 14, 21, and 28 since randomization. Case report form is attached in the Appendix.

3.6 Schedule of Activities

Study day	Screen	Randomization	Day 2	Day 7	Day 14	Day 21	Day 28
Informed consent	X						
Inclusion and exclusion criteria review	X						
Demographics		X					
Coexisting conditions		X					
Onset date and symptoms		X					
Height		X					

Weight		X					
Vital signs and peripheral oxygen saturation (SpO_2)		X		X	X	X	
Respiratory support and parameters		X		X	X	X	
Vasopressors and dosage		X		X	X	X	
Complete blood count		X		X	X	X	
C-reactive protein (CRP) or high-sensitivity CRP		X		X	X	X	
Chemistry panel		X		X	X	X	
Bilirubin		X		X	X	X	
Alanine transaminase (ALT)		X		X	X	X	
Aspartate transaminase (AST)		X		X	X	X	
Creatinine		X		X	X	X	
Lactate dehydrogenase		X		X	X	X	
Arterial blood gas		X		X	X	X	
SARS-CoV-2 specific antibody test		X	X	X	X	X	
SARS-CoV-2 nucleic acid test				X	X	X	
Adverse events		X		X	X	X	X
Clinical status							X
Administer study intervention (IV infusion)		X					
Other medications		X		X	X	X	X

4. Sample Size Estimates

According to the distribution across the six-level ordinal outcome of another study among similar patients[17], we calculated that the initial enrollment of 200 patients would have 80% power to detect an odds ratio of 1.50 for a better outcome category with a one-sided type I error of 0.3. The rationale of choosing a high type I error rate is to prevent enrolling too many patients to an investigational drug that is unlikely to be effective.

5. Statistical Analyses

The efficacy will be analyzed among all randomized participants who received study intervention and provided at least one post-randomization measurement of outcomes. Participants will be analyzed according to the intervention to which they were randomized. (Intention to treat). Safety profile will be analyzed according to the intervention they actually received.

The primary outcome will be analyzed by mixed ordinal logistic regression. Binary outcomes will be compared with the use of mixed logistic-regression model. 28-day mortality and negative

conversion rates of SARS-CoV-2 nucleic acid will be analyzed by Cox proportional-hazards model. Continuous outcomes will be assessed with the use of generalized linear models or general linear models for repeated measures. All models will be adjusted for age, site, and clinical ordinal scale at randomization. P value less than 0.05 will be considered statistically significant. Statistical analyses will be performed by SPSS 22.0.

6. Data and Safety Monitoring Plan

6.1 Benefit/Risk Assessment

Since JS016 is a neutralizing antibody originated from human, off-target binding and tissue cross-reactivity is considered unlikely. Furthermore, the introduction of amino acid substitutions in the fragment crystallizable (Fc) region of JS016 lowered the risk of antibody-dependent enhancement effect and Fc-mediated acute lung injury in animal models[12]. Potential risks of allergic reaction are also considered low based on good tolerability and safety profile of JS016 among healthy volunteers. Given the potent neutralizing activity of JS016 and the lack of effective therapy for COVID-19, the overall assessment of benefit and risk is favorable.

6.2 Protection against Study Risks

All participants will be acknowledged of the aim, process, and potential risks and benefits of the study. Informed consent will be obtained. Due to strict respiratory isolation regulations in participating hospitals, verbal consent will be allowed if approved by ethics committees. The confidentiality of all study participants will be protected. A participant may withdraw from the study at any time at the request of his/her own or representative, but data collected before the withdrawal can be used for analyses.

6.3 Management of Allergic Reactions

Infusions of the study drug will be administered at a controlled rate, and participants will be closely monitored during infusion. Infusing rate will be adjusted or stopped if indicated. Allergic reactions occur during or within six hours after infusion, and include symptoms and signs of, but do not limit to, fever, chills, nausea, headache, bronchospasm, hypotension, angioedema, throat irritation, rash, pruritus, myalgia, and dizziness. The severity of allergic reactions will be evaluated using the Division of Allergy and Infectious Diseases (DAIDS) Table for Grading the Severity of Adult and Pediatric Adverse Events[18] and managed by investigators based on standard clinical practice. If the infusion is definitively discontinued, the participant will remain in the study for follow-up and further evaluations shown in the SoA.

6.4 Adverse Events (AEs) and Serious Adverse Events (SAEs)

An AE is any untoward or unfavorable medical occurrence in a participant, including any abnormal sign, symptom, or disease, temporally associated with their participation in study, whether or not considered related to the study. If a diagnosis is clinically evident (or subsequently determined), the diagnosis, rather than the individual signs and symptoms or lab abnormalities, will be recorded as the AE.
Adverse events are serious if they lead to one of the following outcomes:

- Death
- Life-threatening (i.e., an immediate threat to life)

- Hospitalization or prolongation of hospitalization
- Persistent or significant incapacity or substantial disruption of the ability to conduct normal life functions
- Congenital abnormalities/birth defects
- Other important medical events that may jeopardize the participant and/or may require intervention to prevent one of the outcomes listed above

Severity of AEs will be evaluated according to the DAIDS Table for Grading the Severity of Adult and Pediatric Adverse Events[18].

AEs will be reported by participants, caregivers, or investigators, the last of whom are responsible for documenting, recording, and following up AEs. All AEs and SAEs will be collected from randomization to day 28, and followed up until resolution, stabilization, or loss of follow-up. The DSMB may request to stop enrollment for safety reasons, inform ethics committees, and determine whether it is safe to resume the study.

7. Data and Safety Monitoring

Study supervisors will be responsible for ensuring participants' safety and for reporting AEs to the Ethics Committees and Data and Safety Monitor Board (DSMB).

There will be an independent DSMB who are responsible to evaluate the progress of the study and the quality of data collection, management, and analyses. Another goal of the DSMB is to review interim data and use pre-specified rules to determine the continuing safety and efficacy of the investigational drug. Only masked data will be presented to the DSMB. All data are confidential. Participant identities will be kept confidential unless safety concerns necessitate unmasking some or all data.

Appendix

Case Report Form

Site

□ The First Affliated Hospital of Harbin Medical University
□ The First Hospital of Suihua
□ Shijiazhuang People's Hospital

Demographics and Coexisting Conditions

Initial of name		Gender	□ Male	□ Female
Age		Date of admission		
Height	cm	Weight		kg
Coexisting conditions	□ Diabetes<br>□ Hypertension	□ Coronary heart disease<br>□ Chronic lung disease	□ Malignancy<br>□ Chronic kidney disease	

Disease Onset

Date of symptom onset				
Symptoms of onset	□ Fever	□ Dyspnea	□ Cough	□ Others

Randomization

Date of randomization			Disease severity at randomization	□ Moderate<br>□ Severe
Assignment	□ JS016	□ Control		

Clinical and Laboratory Parameters

	Randomization		Day 2	Day 7		Day 14		Day 21	
Temperature (℃)									
Heart rate (/min)									
Blood pressure (mmHg, systolic/diastolic/median)									
Vasopressor	□ Y	□ N		□ Y	□ N	□ Y	□ N	□ Y	□ N
Drug									
Dosage									
Respiratory rate (/min)									
SpO_2 (%)									
Respiratory support									
□ Nasal cannula<br>Oxygen flow (L/min)									
□ HFNC<br>Oxygen flow (L/min)<br>FiO_2 (%)									
□ NIV<br>FiO_2 (%)<br>EPAP (cmH_2O)									

Repiratory support	Randomization	Day 2	Day 7	Day 14	Day 21
□ IMV<br>FiO_2 (%)<br>PEEP (cmH_2O)					
□ ECMO					
pH					
PaO_2 (mmHg)					
$PaCO_2$ (mmHg)					
HCO_3 (mmol/L)					
White cell count (/μL)					
Lymphocte count (/μL)					
Platelet count (/μL)					
CRP or hsCRP (mg/L)					
Total bilirubin (μmol/L)					
LDH (U/L)					
ALT (U/L)					
Creatinine (μmol/L)					
SARS-CoV-2 IgG					
SARS-CoV-2 IgM					
SARS-CoV-2 CT value					
N gene					
ORF gene					

Adverse Events (from randomization to day 28)

□ Allergic reaction	□ Acute kidney injury	□ Elevated ALT or AST
□ Secondary infection	□ Septic shock	□ Gastrointestinal bleeding
□ Others:		

Medications (from randomization to day 28)

□ Antiviral medication:					
Traditional Chinese Medicine	□ Y	□ N	Glucocorticoids	□ Y	□ N
□ Others:					

Supportive Therapies (from randomization to day 28)

	□ NIV	□ IMV	□ Vasopressor	□ CRRT	□ ECMO
Initiation date					
Termination date					

Clinial outcomes (day 28 since randomization)

□Death	Date of death				
□Discharge	Date of discharge				
□Hospitalized	□ Nasal cannula	□ HFNC	□ NIV	□ IMV	□ ECMO

Reference:

1. Guan W-J, Ni Z-Y, Hu Y, et al. Clinical Characteristics of Coronavirus Disease 2019 in China. New Engl J Med. 2020;382(18):1708-1720.

2. Wang D, Hu B, Hu C, et al. Clinical Characteristics of 138 Hospitalized Patients With 2019 Novel Coronavirus–Infected Pneumonia in Wuhan, China. JAMA. 2020;323(11):1061-1069.

3. Zhou F, Yu T, Du R, et al. Clinical course and risk factors for mortality of adult inpatients with COVID-19 in Wuhan, China: a retrospective cohort study. Lancet. 2020;395(10229):1054-1062.

4. Huang C, Wang Y, Li X, et al. Clinical features of patients infected with 2019 novel coronavirus in Wuhan, China. Lancet. 2020;395(10223):497-506.

5. Ruan Q, Yang K, Wang W, et al. Clinical predictors of mortality due to COVID-19 based on an analysis of data of 150 patients from Wuhan, China. Intensive Care Medicine. 2020;368(2):1-3.

6. Feng Y, Ling Y, Bai T, et al. COVID-19 with Different Severities: A Multi-center Study of Clinical Features. Am J Resp Crit Care. 2020;201(11):1380-1388.

7. Chen N, Zhou M, Dong X, et al. Epidemiological and clinical characteristics of 99 cases of 2019 novel coronavirus pneumonia in Wuhan, China: a descriptive study. The Lancet. 2020;395(10223):507-513.

8. Docherty AB, Harrison EM, Green CA, et al. Features of 20 133 UK patients in hospital with covid-19 using the ISARIC WHO Clinical Characterisation Protocol: prospective observational cohort study. BMJ. 2020;369:m1985.

9. Richardson S, Hirsch JS, Narasimhan M, et al. Presenting Characteristics, Comorbidities, and Outcomes Among 5700 Patients Hospitalized With COVID-19 in the New York City Area. JAMA. 2020;323(20):2052-2059.

10. RECOVERY Collaborative Group et al. Dexamethasone in Hospitalized Patients with Covid-19 — Preliminary Report. New Engl J Med. Published online July 17, 2020.

11. Hoffmann M, Kleine-Weber H, Schroeder S, et al. SARS-CoV-2 Cell Entry Depends on ACE2 and TMPRSS2 and Is Blocked by a Clinically Proven Protease Inhibitor. Cell. 2020;181(2):271-280.e8.

12. Shi R, Shan C, Duan X, et al. A human neutralizing antibody targets the receptor-binding site of SARS-CoV-2. Nature. 2020;584(7819):120-124.

13. www.nhc.gov.cn/yzygj/s7653p/202104/7de0b3837c8b4606a0594aeb0105232b.shtml

14. Beigel JH, Tebas P, Elie-Turenne M-C, et al. Immune plasma for the treatment of severe influenza: an open-label, multicentre, phase 2 randomised study. Lancet Respir Medicine. 2017;5(6):500-511.

15. Wang Y, Fan G, Salam A, et al. Comparative effectiveness of combined favipiravir and oseltamivir therapy versus oseltamivir monotherapy in critically ill patients with influenza virus infection. J Infect Dis. 2019;221(10):1688-1698.

16. Cao B, Wang Y, Wen D, et al. A Trial of Lopinavir–Ritonavir in Adults Hospitalized with Severe Covid-19. New Engl J Med. 2020;382(19):1787-1799.

17. Cavalcanti AB, Zampieri FG, Rosa RG, et al. Hydroxychloroquine with or without Azithromycin in Mild-to-Moderate Covid-19. New Engl J Med. 2020;383(21):2041-2052.

18. Division of AIDS (DAIDS) Table for Grading the Severity of Adult and Pediatric Adverse Events (Corrected Version 2.1 July 2017).
